# Qualitative Study on the Influencing Factors and Countermeasures Against Job Burnout Among Organ Donation Coordinators

**DOI:** 10.3389/fpubh.2020.571514

**Published:** 2020-10-20

**Authors:** Ai-jing Luo, Ze-hua Xu, Ping-ping Cai, Hai-yan He, Ping Mao, Wen-zhao Xie

**Affiliations:** ^1^The Third Xiangya Hospital of Central South University, Changsha, China; ^2^Key Laboratory of Medical Information Research (Central South University), College of Hunan Province, Changsha, China; ^3^School of Life Sciences, Central South University, Changsha, China; ^4^Public Health College of Central South University, Changsha, China; ^5^Ruijin Hospital, Shanghai Jiao Tong University School of Medicine, Shanghai, China

**Keywords:** organ donation coordinators, job burnout, influencing factor, countermeasure, semistructured interview

## Abstract

**Background:** Most organ donation coordinators suffer varying degrees of anxiety, depression and poor sleep caused by constant work pressure, and their professional identity is only at a medium level. All of this leads to a great risk of job burnout.

**Objective:** To identify the influencing factors of and effective countermeasures against job burnout among organ donation coordinators.

**Method:** Semistructured interviews were used for data collection. In-person or phone interviews were conducted from December 2017 to June 2018.

**Results:** 12 organ donation coordinators who came from 7 different provinces and cities in China were interviewed. The interview data were sorted, and relevant topics were extracted and summarized in terms of two aspects, namely, factors that influenced job burnout in organ donation coordinators and effective countermeasures for dealing with job burnout.

**Conclusion:** Factors influencing job burnout among organ donation coordinators include personal factors, job responsibilities, salary and benefit factors, and donor family factors. Measures to help organ donation coordinators effectively address burnout include self-regulation, social support, and positive events.

## Introduction

Although organ donation rate has increased in recent years, organ shortage is still a very serious problem. There is another person added to the transplant waiting list every 10 min and 20 people die each day waiting for an organ transplant (1). Most studies on factors affecting organ donation rates have examined informed consent policies, religious and cultural beliefs, cognition of death, and misunderstandings about donation, and issues related to the application process itself (2–7). However, little attention has focused on the high turnover of organ donation coordinators (8). Organ donation coordinators are professionals who are equipped with specific professional knowledge and travel bestween hospitals in search of prospective patients, namely, organ donors who meet the criteria for brain death, or cardiac death. These professionals, as the link between organ donors and recipients, are also responsible for communicating with these patients' families, who could help to convince the patient to donate his or her organs so that prospective organ donors can more easily become actual organ donors. In 2010, China officially established the professional position of organ donation coordinator. However, due to the influence of China's traditional culture, organ donation after death began only a few years ago. The limited media publicity given to organ donation and organ donation coordinators and the imperfections of the organ donation law result in low public awareness of organ donation coordinators and make it difficult for them to perform their job. At present, most organ donation coordinators suffer varying degrees of anxiety, depression, and poor sleep caused by constant work pressure, and their professional identity is only at a medium level (9, 10). All of this leads to a great risk of job burnout.

Burnout is a response to chronic strain within the workplace characterized by feelings of inefficacy (reduced personal accomplishment), cynicism (depersonalization), and emotional exhaustion (11). Maslach, an American social psychologist, proposed that job burnout is an unhealthy psychological state (12). Jesse et al. (11) performed a national cross-sectional survey of 218 transplant surgeons and found 40.1% reported high levels of emotional exhaustion, 17.1% reported high levels of depersonalization, and 46.5% reported low personal accomplishment. They found that lower decisional authority, lower coworker support, less frequent difficult patient interactions but greater discomfort with difficult patient interactions predicted lower personal accomplishment. Yang et al. found transplant nurses in China experience burnout and older age, being married, and having children may increase the risk of burnout (13). Bury et al. investigated the experience of professional burnout in a large group of Polish transplant coordinators (14). Exhaustion was positively correlated with years of working as a transplant coordinator but not with participants' age (14). Kader et al. (15) investigated the occupational burnout level of 122 organ donation coordinators in Turkey and found that a low level of emotional exhaustion and depersonalization of coordinators and a moderately decreased sense of accomplishment. Unmarried coordinators without medical backgrounds had a lower sense of accomplishment, while coordinators with a college degree had a higher level of depersonalization. Kim et al. (16) investigated 14 coordinators at an organ transplant center in the southeastern United States and found that they had an average level of job burnout; furthermore, the higher the coordinator's education level was, the lower his or her level of job burnout was. Gruener (17) investigated job burnout and its influencing factors among organ donation coordinators in Israel and found that the average job burnout level of coordinators was relatively low; furthermore, job burnout was negatively correlated with a sense of control but positively correlated with job satisfaction and self-realization. However, Kress et al. (8). conducted an online survey of 326 organ donation coordinators and found that ~26% of them were considering quitting and that those who had been working for 2 years had the strongest intention to quit their jobs. Our research group (18) preliminarily investigated 312 organ donation coordinators in China. The questionnaire included the following: the Chinese version of MBI-GS was used to evaluate the burnout levels of organ donation coordinators. We found that the incidence of job burnout was as high as 65.02%. Mild burnout was the main trend, and a decreased sense of accomplishment was the main manifestation (18). For a deeper understanding of the factors influencing job burnout among organ donation coordinators, purposive sampling was adopted. The organ donation coordinators from the preliminary study who had a moderate or severe degree of job burnout were selected as the interviewees. Semi-structured interviews were used for data collection. Colaizzi's seven-step analysis method was used to guide data analysis (19). The research purpose was to identify the influencing factors of and effective countermeasures against job burnout among organ donation coordinators.

## Study Object

### Sample Selection

The inclusion criteria for the samples were as follows: ① organ donation coordinators who had obtained an organ donation coordinator qualification certificate; ② organ donation coordinators with at least 1 year of work experience as an organ donation coordinator; ③ registered and active organ donation coordinators; ④ organ donation coordinators who provided their informed consent to participate in the research. The exclusion criteria were as follows: ① organ donation coordinators who had not been involved in an organ donation coordination in the past year; ② organ donation coordinators who had not independently coordinated an organ donation case; ③ organ donation coordinators who were off-duty for various reasons.

### Determination of Sample Size

Our research group preliminarily investigated 312 organ donation coordinators in China. We found that the incidence of job burnout was as high as 65.02%. Among them, 45.94% had mild job burnout, 16.96% had moderate job burnout, and 2.12% had severe job burnout (18). For a deeper understanding of the factors influencing job burnout among organ donation coordinators, the organ donation coordinators who had a moderate or severe degree of job burnout were selected as the interviewees. In qualitative research, the sample size depends on whether the data obtained reaches saturation. If new interviewees continue to be included but no new information is obtained, the data are said to have reached saturation, and sampling is complete. A total of 12 interviewees were selected for this study.

### Research Tools

An interview outline was designed according to the relevant literature and our previous survey. Through consultations with two experts in psychology and preliminary interviews with two organ donation coordinators, we made some proper adjustments and then the final version of the interview outline was determined (Table 1). In our previous survey, when analyzing the results of the job satisfaction of coordinators, we found that the coordinator had the lowest score in the promotion dimension and work process dimension (18). This indicated that the coordinator had the lowest satisfaction with promotion and work process. Therefore, when the coordinator mentions these two factors again, we can use follow-up methods to conduct in-depth discussions to improve the reliability of the research results.

**Table 1:** Interview guide.

1. How do you become an organ donation coordinator?
2. What does being an organ donation coordinator bring? Like sense of accomplishment?
3. Did you experience burnout as a coordinator? What caused it?
4. What did you do to reduce the feeling of burnout (Psychologically and operationally)? Is it effective?
5. Have you ever thought of quitting the position of coordinator? Or have you tried seeking other positions?
6. What do you think of the working environment of the organ donation coordinator?
7. What do you think of your daily work?(Working time, workload, work process, work pressure, and sources, how to deal with the relationship between work and life)
8. Do you think your current job promotion opportunities are great? What are the reasons?
9. What do you think of the salary and benefits of the coordinator?
10. How do you get along with your colleagues and leaders?
11. What do your family and friends think about your current job as an organ donation coordinator?
12. In the process of coordinating organ donation, when you encounter unsatisfactory or urgent matters, how do you usually solve it (Including psychological and operational)? Is it effective?
13. When you encounter emergencies or difficulties in the process of coordinating organ donation, what kind of help do you most hope for? (Material assistance, spiritual assistance, legal and policy support related to organ donation, authorization from the state, and government)
14. What things do you think will affect your motivation at work?
15. Do you have any suggestions or expectations for the work of the organ donation coordinator?

### Data Collection

#### Data Collection

In-person or phone interviews were conducted from December 2017 to June 2018. The semi-structured interviews with organ donation coordinators were conducted in Hunan Province, China. The interviewers contacted the interviewees in advance. Informed consent was obtained from the interviewees. Specific details such as the time and venue for interviews were determined to ensure the quality of the interview. Before the interview, the interviewers asked the interviewees whether they preferred for the interview to be recorded with a recording pen or by note-taking. During each interview, one interviewer was responsible for communicating with the interviewee. Another interviewer was responsible for recording the whole process and providing reminders about the progress of the interview.

#### Data Sorting and Analysis

Within 2 days after the interview, two interviewers performed open coding independently to generate the initial codes. These independently generated codes were discussed among all authors in group sessions, with differences reconciled through negotiated consensus and repeated consideration of our research questions. Colaizzi's seven-step analysis method was used to extract relevant content from the interview data.

### Ethics

Approval for the study was obtained from the Institutional Ethics Committee of the Third Xiangya Hospital, Central South University.

## Results

### General Information About the Interviewees

In this study, after interviewing 12 subjects, no new interview information was found, indicating that the data had reached saturation. Therefore, the sample size of the qualitative study was 12. These 12 subjects were numbered D1, D2,…, D12. They came from 7 different provinces and cities in China. Their general information is shown in Table 2.

**Table 2:** General information of the interviewees (*n* = 12).

**Number**	**Gender**	**Age**	**Education**	**Marital status**	**Years of service**	**Years of service as coordinator**	**Job type**	**Workplace**
D1	Female	31	Master's degree	Married	10	3	Part-time	Hospital
D2	Male	60	Associate degree	Married	44	1	Full-time	The red cross
D3	Female	34	Master's degree	Married	15	4	Full-time	The red cross
D4	Female	28	Bachelor's degree	Single	6	2	Part-time	The red cross
D5	Female	31	Master's degree	Married	12	3	Part-time	Hospital
D6	Female	49	Bachelor's degree	Married	31	3	Full-time	The red cross
D7	Male	38	Bachelor's degree	Married	21	2	Part-time	The red cross
D8	Female	32	Bachelor's degree	Married	13	7	Full-time	Hospital
D9	Male	36	Master's degree	Married	11	6	Full-time	Hospital
D10	Female	50	Associate degree	Married	19	11	Full-time	The red cross
D11	Male	44	Master's degree	Married	19	8	Full-time	The red cross
D12	Male	34	Bachelor's degree	Married	12	11	Full-time	Hospital

### Interview Results

The interview data were sorted, and relevant topics were extracted and summarized in terms of two aspects, namely, factors that influenced job burnout in organ donation coordinators and effective countermeasures for dealing with job burnout. The results are shown in Tables 3, 4. Details are shown below.

**Table 3:** Factors influencing job burnout in organ donation coordinators.

**Category**	**Subcategory**
Personal factors	(1) Original intention of working as an organ donation coordinator (2) Professional background (3) Psychological quality
Job responsibilities	(1) Work hours (2) Workload (3) Sense of responsibility (4) Failure cases
Promotion and salary and benefits	(1) Professionalization (2) Salary and benefits
Donor family factors	(1) Recognition by the donor's family (2) Grief from the donor's family

**Table 4:** Countermeasures taken by organ donation coordinators against job burnout.

**Category**	**Subcategory**
Self-regulation	(1) Allowing time for rest and relaxation (2) Self-comfort (3) Empathy (4) Be rational about the work
Social support	(1) Social recognition (2) Support from family and friends (3) Support from superiors and peers at work (4) Seeking help from psychological counselors
Positive events	(1) Successful cases (2) Recognition from the donor's family

#### Factors Influencing Job Burnout in Organ Donation Coordinators

Topic 1: Personal factors.

(1) The original intention of working as an organ donation coordinator
  The interviews showed that many organ donation coordinators did not sign up for the job out of their desire. They started in the role due to work needs or as a result of orders from their superiors.
  “*The reason for becoming a coordinator is the demand for the line of work. I was assigned this job by my boss, who arranged for me to participate in the national training program. After I completed the training, I was asked to become a coordinator.” (D7)*
  Five of the interviewees said that they did not accept the job of their own free will. They showed a strong intention to quit their jobs and a high degree of job burnout, which affected the stability of the team of organ donation coordinators.
  “*I'm a doctor, and it's a personal loss for me to do this job. I've thought about quitting 10,000 times. However, within the (organ transplant) team, I have to follow orders from my boss.” (D9)*
(2) Professional background
  For those organ donation coordinators who had a medical- or psychology-related professional background, it was less difficult to carry out their work. Their rate of successful case coordination was higher than that of organ donor coordinators without such backgrounds, and their work pressure was comparatively low.
  “*If you have a medical background, it is better. Without a medical background, it is difficult to do your job, and it is easier for you to get frustrated.” (D7)*
(3) Psychological quality
  The coordinators with poor mental endurance did not know how to self-regulate when they encountered difficulties and were prone to job burnout.
  “*The coordinator's mental endurance is very important. If he/she does not have certain work experience and has poor receptivity, he/she will encounter emotional breakdown when he/she is bombarded with questions from the patient's family members, who might have a hard time understanding.” (D7)*
  “*I don't think I'm suitable for this job because I'm not emotionally strong, and I'm susceptible to the grief of patients' families, and I have to witness the whole process of organ donation, including organ removal and wound suture. When rigor mortis sets in, I feel very, very uncomfortable.” (D5)*

Topic 2: Job responsibilities.

(1) Work hours
  Organ donation coordinators are on call 24/7, with no regular breaks or holidays, and most cases are coordinated in the middle of the night. Many coordinators feel exhausted.
  “*What impresses me the most about this job is that I have to leave for another case when I just came back from a long-distance business trip and am getting ready for some sleep. I feel as if I have to keep working 24 hours without a break. I feel that I don't have time for anything else.” (D1)*
  “*More often than not, coordinators have to do their job at night. They will feel exhausted if they have to stay up late and work at night often.” (D4)*
  “*The work schedule of a coordinator is not fixed. They may have to go to work during the daytime or at night. They must keep their cellphones turned on all the time. They are under enormous stress.” (D12)*
(2) Workload
  Within the organ transplant team, organ donation coordinators are responsible for the majority of the work during the organ donation process. The heavy workload exhausts them. In addition, coordinators often bear many responsibilities during this process, which leads to great psychological pressure.
  “*The main reason that I'm tired is the workload. For each case, you have to repeatedly answer all the questions raised by the patient's family, and you have to be patient with them. You are also supposed to try your best to meet the conditions and requirements raised by each family member.” (D1)*
  “*I think that within the team, most of the work is done by the coordinator, who has to take on a lot of responsibilities and risks. All of this makes me feel stressed. The stress comes mainly from these responsibilities, and this job is about a matter of life and death. If anything goes wrong during this process, the coordinator has to take the blame.” (D5)*
(3) Sense of responsibility
  Four interviewees indicated that their job pressure mainly came from their sense of responsibility. If this kind of pressure could not be effectively alleviated, job burnout and resignation intention would occur.
  “*We haven't completed a single case in a while, and we haven't even got much information, so we feel a lot of pressure. My boss does not pressure me in any way, yet I just feel pressured out of nowhere.” (D8)*
(4) Failure cases
  Failure cases undermined the self-confidence of the organ donation coordinators, leading to a sense of frustration and a diminished sense of personal accomplishment.
  “*If you don't succeed in two or three cases, it's hard for you to continue doing it with 100% confidence and interest. Once you are affected by the failure of a case, you will try so hard to succeed in the next case. The pressure you have to face during this process is tremendous.” (D7)*

Topic 3: Promotion and salary and benefits.

(1) Professionalization
  Almost every interviewee expressed that the profession of organ donation coordinators lacks normalization, professionalization, promotion mechanisms, and incentive mechanisms. Hence, coordinators are not motivated. They see no future. It is easier for them to experience job burnout and have the intention to resign. The professionalization of organ donation coordinators could enhance their professional identity and their passion for this job.
  “*The problem of promotion channels has a big impact on me. If I'm still a coordinator in another ten years, I will feel that I have accomplished nothing.” (D9)*
  “*In a large sense, the job burnout of coordinators mainly comes from the fact that the country does not pay enough attention to the job and does not lay out a clear career path for coordinators. Thus, coordinators do not have any goals for the future. They have no future career prospects.” (D11)*
(2) Salary and benefits
  During the interviews, six organ donation coordinators mentioned problems with their salaries, which they hoped that the state could solve as soon as possible. However, coordinators' salaries and benefits standards are not stipulated at the national level. Many of the coordinators believed that they work a lot but are paid little. This, to a certain extent, affects job satisfaction among coordinators and causes them to experience job burnout.
  “*We have lost many coordinators, which is mainly because of the salary issue.” (D6)*
  “*In our unit, the income of the coordinator is relatively low. It barely makes ends meet, so there must be an impact (on job burnout).” (D12)*

Topic 4: Donor family factors.

(1) Recognition by the donor's family
  Recognition by and trust from the donor's family is a prerequisite for allowing the organ donation coordinator to carry out their work smoothly and directly affects the success of the case. Distrust and doubt from the donor's family are a major source of stress for organ donation coordinators that diminishes their passion for work and their sense of accomplishment.
  “*Some family members do not understand what you are doing, and some of them may even verbally attack you. Then, you don't even get a chance to explain. It can be very stressful. Some will ask for your work permit and your ID card before you communicate with them, which will make you feel very stressed and feel that your sincerity is not recognized.” (D1)*
(2) Grief from the donor's family
  Grief from the donor's family exerts a great impact on coordinators. The coordinator is the witness of the donor's organ donation and death. As a result, coordinators are in a state of constant stress and psychological pressure.
  “*I've been through so many cases. I probably shed tears in 50 to 60 cases of mine. Especially the children… I shed tears for children most of the time. Parents cry their hearts out seeing their children dying. It makes you cry too. Therefore, I think the psychological pressure may also come from these aspects.” (D6)*

#### Countermeasures Taken by Organ Donation Coordinators Against Job Burnout

Topic 1: Self-regulation.

(1) Allowing time for relaxation
  Proper relaxation may help organ donation coordinators relieve stress and self-regulate their mental state.
  “*When I'm in a bad mood, I try to think about happy things. I will go to eat, drink and entertain myself. Then, I'll be okay.” (D4)*
  “*When I'm stressed, I watch movies and get exercise.” (D7)*
(2) Self-comfort
  Since the position of organ donation coordinator in China has been around for only a short time, many people are not clear about the nature and responsibilities of the work. Some coordinators expressed that when they confided in others, they were not always understood and supported. Therefore, coordinators mainly rely on self-comfort to alleviate their stress.
  “*It's always helpful to comfort yourself when something doesn't go your way. After all, it's just a job you do. When I encounter stress, I rely on self-comfort and self-regulation.” (D7)*
(3) Empathy
  Empathy can significantly reduce the stress of organ donation coordinators. The respondents indicated that putting themselves in the shoes of the donor's family enabled them to handle any case properly, even failure cases.
  “*As a coordinator, I consider some practical issues by standing in the position of the donor's family whenever I try to communicate with them. It is all right, even if I fail a case.” (D8)*
(4) Be rational about the work
  The organ donation coordinators indicated the importance of having a proper understanding of the work and approaching the dissatisfying part of the job rationally. They also recommended keeping work and life separate to reduce the impact of negative events at work that organ donation coordinators may experience.
  “*Sometimes, I may complain about my work and lose my temper on the way back home from a business trip, but I don't usually bring this state of mind back home. I tell myself that work and life are separate.” (D1)*
  “*At first, I felt depressed, but now I think it is okay as long as I have tried my best. If I try to explain the significance of organ donation to the donor's family, but they still don't agree to donate, I'll just have to give up. I am not as stressed as before.” (D8)*

Topic 2: Social support.

(1) Social recognition
  Media publicity may help the public to understand organ donation and organ donation coordinators and may improve the social recognition of coordinators. Such results may make it easier for coordinators to gain the understanding and help of the public and obtain happiness from work.
  “*The media here pay more attention to organ donation. Most likely because I have done a lot in this area, I often get interviewed by the media. Like one time, when I was taking a bus, the driver said that all the buses should not allow me to pay bus fares because I work for the public. If you have the trust, the approval, the motivation, and the understanding, why would you still feel tired?” (D10)*
(2) Support from family and friends
  More than half of the coordinators said that support and recognition from their family and friends motivate them during their work as coordinators and help them reduce the impact of negative emotions.
  “*It's important to have the support of your family and friends. Their recognition of your work makes you want to do it. I think this helps reduce the level of job burnout.” (D8)*
  “*There are so many good-hearted people, friends around me, friends I know or do not know… They understand me, support me and trust me. In the face of so many beautiful things and so much positive energy, I won't feel tired.” (D10)*
(3) Support from superiors and peers at work
  Support from superiors can help coordinators solve problems, reduce stress, and increase their passion for their work.
  “*Your employer's support and approval of your work may have a big impact on job burnout. If your boss in your workplace does not support you and recognize your work, you certainly do not want to do this.” (D8)*
  *Peer support mainly refers to the cooperation and support of relevant hospitals and departments, who are the main sources of information about potential donors. These hospitals and departments also serve as the main venues for communication and coordination between coordinators and patients. Therefore, peer support may increase the professional identity of coordinators and reduce the degree of job burnout*.
  “*The support and cooperation of the medical institutions and the provision of meeting rooms will affect coordinators' passion for work.” (D7)*
(4) Seeking help from psychological counselors
  Some of the coordinators, when troubled by psychological problems, sought help from psychological counselors at the psychological association. This method proved to be effective.
  “*When I feel tired, or when I don't want to do it, I go to talk to the professionals at the local psychological association, who hold qualifications for psychological counseling. Talking to them sets your heart at ease.” (D3)*

Topic 3: Positive events.

(1) Successful cases
  Successful cases and the recipient's restoration to health could increase the coordinators' sense of happiness and accomplishment.
  “*The patient's recovery has a positive effect on me.” (D4)*
  “*When the coordinated case is successful, the patient gets a donor organ, and the surgery is successful, I feel a great sense of accomplishment, and I think this job is so meaningful. Whenever I might experience job burnout, I think of this aspect of work and become cheerful.” (D8)*
(2) Recognition from the donor's family
  Recognition from the donor's family has a positive impact on coordinators, especially when the family regards the coordinator as a family member. This can enhance the coordinator's professional identity and significantly reduce his/her job burnout.
  “*Some families will consider you one of their relatives because you are the only bridge between the donor and the recipient. Through you, the donor's family may get some information about the recipient. They will be especially kind to you, and afterward, they will still be grateful for your work. Therefore, this kind of family recognition, I think, may also reduce the level of job burnout to some extent.” (D1)*

## Discussion

### Analysis of Factors Influencing Job Burnout Among Organ Donation Coordinators

Through qualitative research, this study found that the factors influencing job burnout in organ donation coordinators include personal factors, job responsibilities, salary and benefit factors, and donor family factors. This finding is similar to the results of relevant studies on job burnout within and outside China (9, 10).

#### Personal Factors

Such factors mainly include the intention, professional background, and psychological quality of organ donation coordinators. Our conclusions are similar to those of Henriksen et al. (20). Organ donation coordinators who embark on this line of work in response to an order from their superiors instead of in response to their wishes have less passion and low efficiency when doing their job. They tend to feel exhausted and express a desire to leave the post. Different professional backgrounds also have an impact on job burnout among organ donation coordinators, which is consistent with Kader's conclusion (15). Perhaps because organ donation coordinators with a medical background often have the experience and skills to communicate with patients and their families, their words are more persuasive during their communication with potential donors' families. Organ donation coordinators with good psychological qualities will face all manner of difficulties with a positive attitude and make full use of available resources to resolve the difficulties and achieve their goals. Such behaviors help coordinators make full use of their strengths and prevent the occurrence of job burnout.

It is suggested that when employing organ donation coordinators, relevant departments should make the work responsibilities of the position open and transparent, fully respect the will of individuals, and avoid coercion. In addition, it is recommended that coordinators be selected from among those with a certain professional background. Finally, when recruiting talent, it is advised that psychological tests be conducted to locate candidates with positive psychological qualities that are conducive to the reduction of job burnout.

#### Job Responsibility Factors

Such factors mainly include the work hours, workload, work responsibility and failure cases of the organ donation coordinator. Among these factors, the impact of work time on job burnout was mentioned most often throughout the interviews. The reason for the emphasis on this factor may be that the working hours of organ donation coordinators are not fixed, and coordinators must be on standby 24 h a day, 7 days a week. This condition may lead to irregular routines and physiological problems. In addition, many organ donation coordinators are part-time medical staff who also need to work shifts in hospitals. Doing two jobs at the same time leads to severe physical exhaustion, which prevents the coordinators from devoting themselves to coordinating cases.

The establishment of a flexible time-off mechanism is recommended. Such a mechanism would ensure that coordinators, especially part-time coordinators, have plenty of time to rest, which will reduce the level of job burnout and improve their work efficiency.

#### Salary and Benefit Factors

These factors include professionalization and salary/benefits standardization for organ donation coordinators. As organ donation coordinators in China have not yet obtained professionalization (21, 22), their career prospects are unclear, which may be responsible for their low level of professional identity and lack of a sense of belonging. Moreover, the lack of promotion and incentive mechanisms leads to an absence of rank among organ donation coordinators. Coordinators feel no need to compete in their daily work, and thus they are prone to job burnout. Acceleration of the professionalization of organ donation coordinators is recommended to reduce job burnout. The interviewees pointed out that the salary and benefit problem was the most important reason for the loss of coordinators. Increasing the salaries and benefits of organ donation coordinators will greatly improve their job satisfaction and prevent job burnout.

#### Donor Family Factors

The main task of organ donation coordinators is to communicate and coordinate with potential donors' families. During the entire donation process, coordinators have the most contact with the donors' families and are highly susceptible to the influence of these families. These coordinators' compassion fatigue increases with the accumulation of cases. Studies have found (23) that the higher the level of compassion fatigue, the more likely an individual is to experience job burnout, which harms his or her physical and mental health. Job burnout among organ donation coordinators should be reduced through measures that reduce their compassion fatigue.

### Analysis of Measures Taken by Organ Donation Coordinators to Cope With Job Burnout

Through the interviews, the current study found that the effective measures that organ donation coordinators use to deal with job burnout mainly include self-regulation, social support, and positive events.

#### Self-Regulation

Organ donation coordinators mainly self-regulate by giving themselves time to relax and rest; resorting to self-comfort; feeling empathy, and adopting a rational attitude toward work. This ability to recover from the stressful environment through self-regulation is called psychological resilience (24). Individuals with high psychological resilience can adapt well to adversity and adopt a positive attitude as a coping method, thus maintaining their own physical and mental health (25). A previous study confirmed (18) that individual psychological resilience is significantly correlated with job satisfaction. Therefore, improving the psychological resilience of organ donation coordinators may effectively prevent job burnout.

Great importance should be attached to the cultivation of psychological resilience by organ donation coordinators to improve their adaptability and ability to cope in the face of adversity. Specifically, such efforts should begin with the following factors: First, organ donation coordinators should be given time to relax and rest so that they can engage in self-regulation. Meanwhile, they should be encouraged to cultivate their interests and hobbies in their spare time. Next, psychological training courses should be established for coordinators to turn to for self-distraction. Third, coordinators should be encouraged to take a rational approach to their jobs by distinguishing work from life. Fourth, learn empathy. Fifth, coordinators should receive support in developing self-comfort skills. Finally, coordinators' should be helped to develop a sense of responsibility.

#### Social Support

The types of social support that organ donation coordinators receive include social recognition, support from family and friends, support from peers and superiors, and help from psychological counselors. The establishment of social support systems may help organ donation coordinators to effectively alleviate their stress and reduce job burnout. This finding is similar to that of Choi et al. (26). in a study on nurses. Public recognition helps to enhance professional identity among organ donation coordinators so that they can maintain their passion for work. Support from all sides may provide a good family environment and work atmosphere for coordinators. In case of trouble, coordinators may alleviate their stress by confiding to their family and friends; they may also try to solve their problems by seeking help from their peers and superiors at work. In this way, they can maintain a positive and optimistic attitude toward difficulties and reduce their psychological stress.

#### Positive Events

Positive events mainly refer to successful donation cases and recognition from donors' families. The main job of organ donation coordinators is to promote the transformation of potential organ donors into actual organ donors. The success of donation cases can significantly improve coordinators' sense of happiness and personal accomplishment. In addition, recognition from donors' family members can improve coordinators' passion for work and their self-confidence, which becomes their source of strength. In addition, positive events can enhance the professional identity of organ donation coordinators and increase their willingness to continue in this line of work.

Experience exchange activities should be organized for organ donation coordinators so that they can share in the successful cases of elite coordinators. Such efforts are conducive to improving organ donation coordinators' self-confidence and professional identity.

## Limitations

As well as the significant contributions of the study, there are also some limitations that should be noted. The sample size of our study is small though we had good results. In addition, we mainly use two members of the research group to manually code the interview resources, without using software such as Nvivo to generate code. And future research could focus on intervention study to find out how to reduce the job burnout rate of organ donation coordinators.

## Conclusion

Factors influencing job burnout among organ donation coordinators include personal factors, job responsibilities, salary and benefit factors, and donor family factors. Measures to help organ donation coordinators effectively address burnout include self-regulation, social support, and positive events.

## Data Availability Statement

The datasets generated for this study are available on request to the corresponding author.

## Ethics Statement

Approval for the study was obtained from the Institutional Ethics Committee of the Third Xiangya Hospital, Central South University.

## Author Contributions

WX and AL designed this research and wrote the manuscript. WX, ZX, PC, HH, and PM collected and analyzed the data. WX, ZX, and PC did the literature search. HH provided the support for complete the manuscript. WX and HH revised the paper.

## Conflict of Interest

The authors declare that the research was conducted in the absence of any commercial or financial relationships that could be construed as a potential conflict of interest.
